# Parathyroid hormone, but not vitamin D, is associated with the metabolic syndrome in morbidly obese women and men: a cross-sectional study

**DOI:** 10.1186/1475-2840-8-7

**Published:** 2009-02-03

**Authors:** Jøran Hjelmesæth, Dag Hofsø, Erlend T Aasheim, Trond Jenssen, Johan Moan, Helle Hager, Jo Røislien, Jens Bollerslev

**Affiliations:** 1 Morbid Obesity Center, Vestfold Hospital Trust, Health Region South, Tønsberg, Norway; 2 Hormone Laboratory, Department of Endocrinology, Aker University Hospital, Oslo, Norway; 3 Department of Medicine, Rikshospitalet University Hospital, University of Oslo, Oslo, Norway; 4 Institute of Clinical Medicine, University of Tromsø, Tromsø, Norway; 5 Department of Radiation Biology, The Norwegian Radium Hospital, Oslo, Norway; 6 Department of Clinical Chemistry, Vestfold Hospital Trust, Tønsberg, Norway; 7 Department of Biostatistics, Institute of Basic Medical Sciences, University of Oslo, Oslo, Norway

## Abstract

**Background:**

The prevalence of vitamin D insufficiency and secondary hyperparathyroidism is high among morbidly obese subjects. Further, low serum levels of 25-hydroxyvitamin D (25 [OH]D) and magnesium have been associated with increased risk of the metabolic syndrome (MS), and recently, a possible link between PTH and MS has been reported. Although it is well known that the synthesis and secretion of PTH is regulated by serum levels of calcium, phosphate, magnesium and 25(OH)D, less is known about the possible clustered affiliation of these parameters with MS. We aimed to explore whether MS is associated with abnormal serum levels of PTH, 25(OH)D and magnesium in a population of morbidly obese patients.

**Methods:**

Fasting serum levels of 25(OH)D, PTH and magnesium were assessed in a cross-sectional cohort study of 1,017 consecutive morbidly obese patients (68% women). Multiple logistic regression analyses were used to assess the independent effect of PTH, 25(OH)D and magnesium on the odds for MS (National Cholesterol Education Program [NCEP]) after adjustment for confounding factors.

**Results:**

Sixty-eight percent of the patients had MS. Patients with MS had lower mean serum magnesium (P < 0.001) and higher mean PTH (P = 0.067) than patients without MS, whereas mean 25(OH)D did not differ significantly. Patients with PTH levels in the second to fourth quartiles had higher odds of prevalent MS (odds ratio 1.47 [95% CI 0.92–2.35], 2.33 [95% CI 1.40–3.87] and 2.09 [95% CI 1.23–3.56], respectively), after adjustment for 25(OH)D, magnesium, calcium, phosphate, creatinine, age, gender, season of serum sampling, BMI, current smoking, albuminuria, CRP, insulin resistance and type 2 diabetes. Further, PTH was significantly correlated with systolic and diastolic pressure (both P < 0.001), but not with the other components of MS. The levels of 25(OH)D and magnesium were not associated with MS in the multivariate model.

**Conclusion:**

The PTH level, but not the vitamin D level, is an independent predictor of MS in treatment seeking morbidly obese Caucasian women and men. Randomized controlled clinical trials, including different therapeutic strategies to lower PTH, e.g. calcium/vitamin D supplementation and weight reduction, are necessary to explore any cause-and-effect relationship.

## Background

The metabolic syndrome (MS) is a clustering of risk factors including abdominal obesity, insulin resistance, dyslipidaemia, hyperglycemia, and elevated blood pressure [1]. MS leads to increased risk of diabetes and cardiovascular disease [2]. A number of studies indicate that there is a relationship between 25-hydroxyvitamin D (25 [OH]D), calcium, insulin resistance and MS [3-6]. However, in few of these reports the findings are adjusted for confounding variables with putative associations to MS. Parathyroid hormone (PTH) and magnesium are of particular interest in this context, as elevated levels of PTH and hypomagnesemia have been reported together with MS [7-9]. Low serum magnesium levels have also been linked to insulin resistance [10] and increased risk of type 2 diabetes [11].

Although it is well known that the synthesis and secretion of PTH is tightly regulated by serum levels of calcium and phosphate, serum concentrations of vitamin D and magnesium do also influence PTH levels [12,13]. However, less is known about the possible clustered affiliation of these parameters with MS. Postmenopausal women with normal serum calcium and creatinine but inappropriately high PTH, had higher serum glucose, triglycerides and BMI, but lower HDL-cholesterol concentrations than controls [14]. Increasing PTH levels seem to be associated with MS in older men, but not in women and younger men [8,9]. In contrast, no significant relationship between PTH, vitamin D and MS has been shown in morbidly obese subjects [15].

The objective of the present study was to explore whether MS is associated with abnormal serum levels of PTH, vitamin D and magnesium in a large cohort of morbidly obese patients.

## Methods

### Study population, data collection and ethics

A total of 1,170 consecutive morbidly obese patients who attended a tertiary care center between November 28, 2005 and September 16, 2008, were considered for inclusion. After the exclusion of 153 subjects due to previous bariatric surgery (n = 38), non-Caucasian ethnicity (n = 31), estimated glomerular filtration rate (*eGFR*)<60 ml/min/1.73 m^2 ^(n = 42), type 1 diabetes (n = 12), serum calcium > reference range (2.53 mmol/l; n = 15), or lack of data necessary to diagnose MS (n = 18), a total of 1,017 Caucasian morbidly obese patients were included in the analysis.

The study was approved by the Regional Committee for Medical Research Ethics (S-05175) and was performed in accordance with the Declaration of Helsinki [16].

### Physical examination

Weight and height were measured in patients wearing light clothing but no shoes, and BMI was calculated (kg/m^2^). Waist circumference (WC) was measured at the level midway between the lowest rib margin and the iliac crest. Blood pressure was measured with an appropriately sized cuff after at least 5 minutes rest with the patient seated in an upright position. Three measurements were made and the average of the second and third measurement was registered and used in the analyses.

### Definitions

Type 2 diabetes was diagnosed in patients who had a prior history of type 2 diabetes or a fasting serum glucose level ≥ 7.0 mmol/l [17]. Patients with previously diagnosed hypertension, and patients with blood pressure ≥ 130/85 mmHg [1], were categorized as having elevated blood pressure. MS was diagnosed in patients with at least 3 of the following characteristics [1]; Elevated WC (≥ 102 cm in men and ≥88 cm in women), elevated fasting triglycerides (≥1.7 mmol/l), elevated blood pressure (see above), elevated fasting glucose (≥ 5.6 mmol/l) or diabetes, or reduced HDL-cholesterol (< 1.0 mmol/l in men and <1.3 mmol/l in women). Homeostasis Model Assessment Insulin Resistance (HOMA- IR) was calculated as ([fasting serum glucose (mmol/l) * fasting serum insulin (pmol/l)]/135) [18]. The *eGFR *was calculated with the MDRD equation; eGFR = 30849 * serum creatinine^-1.154 ^* age^-0.203 ^* 0.742 (if female) [19]. Albuminuria was defined as present if the albumin/creatinine ratio was ≥ 2.5 mg/mmol or ≥ 3.5 mg/mmol, in men and women, respectively [20].

Vitamin D sufficiency, insufficiency, and deficiency were defined as serum 25(OH)D concentrations (nmol/L) > 75, < 50, and < 25, respectively [21]. Secondary hyperparathyroidism was diagnosed in patients with a serum PTH concentration > 6.9 pmol/L and serum calcium < 2.54 mmol/l (upper limits of reference ranges).

### Laboratory analyses

Blood samples were obtained after an overnight fast by venipuncture in vacutainer gel tubes, and serum was separated from cells within 2 hours. Analyses of serum glucose, creatinine, calcium, albumin, magnesium, CRP and blood lipids were performed using dry reagent slide technology on the Vitros 950 Analyzer until November 2006 and Vitros FS 5.1 thereafter (Ortho-Clinical Diagnostics, New York, USA). Intact PTH was assayed using an electrochemiluminescence immunoassay on the Elecsys 2010 (Roche Diagnostics GmbH). The coefficients of variation for magnesium and PTH were 2% and 6%, respectively. Glycosylated hemoglobin was analyzed by high performance liquid chromatography (HPLC) using Tosoh HLC-723 G7 (Tosoh Corporation, Tokyo, Japan). Serum calcium was adjusted for albumin; serum calcium = (serum calcium [measured] - 0.018 * [serum albumin - 42]). Sera for analysis of Insulin and 25(OH)D were stored at -20°C and analysed within 1 week of blood sampling at the Hormone Laboratory, Aker University Hospital. Insulin and 25(OH)D (the sum of 25-hydroxyvitamin D_2 _and 25-hydroxyvitamin D_3_) were analyzed in serum by radioimmunoassay (Linco Research Inc, St. Charles, MO, and DiaSorin, Stillwater, MN). The interassay coefficients of variation for insulin and 25(OH)D were 8% and 14%, respectively. All other analyses were performed on the day of blood sampling at Department of Clinical Chemistry at Vestfold Hospital Trust.

### Statistical analysis

Data are given as mean (SD) or proportions unless otherwise stated. Skewed data were ln-transformed to approximate normality before statistical analyses when needed. Differences between groups were analyzed using one way analysis of variance or independent samples *t*-test for continuous data and χ^2 ^for categorical data. Spearman's correlation analysis was used to assess bivariate correlations between continuous variables. Multiple logistic regression with predefined explanatory variables was used to assess odds for MS. We fitted three separate multiple logistic regression models for MS. First (model 1), serum PTH and parameters known to be involved in its regulation; 25(OH)D, calcium, phosphate, magnesium and creatinine, were entered in a multiple logistic regression analysis with MS (yes/no) as the dependent variable. Second (model 2), other confounding factors such as age, gender, season of blood sampling, BMI, current smoking, albuminuria, CRP and HOMAIR were added to model 1. Third, (model 3), presence or absence of type 2 diabetes was added to model 2. Finally, PTH was divided into quartiles, and this new variable replaced PTH as a continuous variable in model 3. A 5% statistical significance level was chosen. The analyses were implemented using SPSS 16.0 (SPSS, Chicago, IL).

## Results

### Characteristics according to presence or absence of MS

Clinical and biochemical characteristics of the 1,017 included patients are shown in table 1. Sixty-eight percent of the patients had MS, 43% had MS but not type 2 diabetes, and 25% had both MS and type 2 diabetes. Approximately two thirds of the patients were female. All men and women had a WC >102 cm and >88 cm, respectively. On average, patients with MS were 5 years older, and they had a higher prevalence of coronary heart disease, diabetes and hypertension, than patients without MS (table 1). The proportions of patients with vitamin D insufficiency and secondary hyperparathyroidism were high, 51% and 24%, respectively, but did not differ significantly between groups.

**Table 1:** Characteristics of patients according to absence or presence of metabolic syndrome

		Metabolic syndrome		
Variables	Total	Absent	Present	P
*n*	1017	323 (32%)	694 (68%)	
Women	68%	73%	65%	0.008
Age (years)	42 (12)	39 (12)	44 (12)	<0.001
Type 2 diabetes	26%	1.5%	37%	<0.001
Coronary heart disease	4.3%	0.9%	5.9%	0.001
Elevated blood pressure	70%	40%	83%	<0.001
Current smoking	26%	27%	26%	0.735
BMI (kg/m^2^)	44.7 (6.2)	44.2 (6.1)	45.0 (6.2)	0.079
Waist (cm)	133 (14)	130 (15)	134 (14)	<0.001
**PTH, vit D and magnesium**				
PTH (pmol/l)	5.8 (2.3)	5.6 (2.3)	5.9 (2.4)	0.067
25(OH)D (nmol/l)	52 (22)	53 (20)	52 (23)	0.127
Magnesium (mmol/l)	0.84 (0.08)	0.86 (0.07)	0.84 (0.08)	<0.001
Calcium (mmol/l)	2.35 (0.07)	2.35 (0.06)	2.36 (0.07)	0.392
Phosphate (mmol/l)	1.09 (0.17)	1.09 (0.15)	1.10 (0.17)	0.517
Creatinine (μmol/l)	65 (11)	65 (10)	66 (12)	0.889
Vitamin D supplement use	11%	11%	11%	1.000
**HbA1c and insulin resistance**				
HbA1c	6.0 (1.3)	5.3 (0.4)	6.3 (1.4)	<0.001
HOMA-IR	6.3 (6.0)	3.9 (2.3)	7.5 (6.8)	<0.001
**Inflammation**				
CRP (≥ 7 mg/l)	69%	67%	70%	0.400
**Drugs**				
Thiazides	14%	7%	17%	<0.001
Loop diuretics	9%	4%	11%	<0.001
Angiotensin II Type 1 Receptor	8%	3%	10%	0.001
Blocker or ACE-inhibitor use				
**Albuminuria**	11%	4%	14%	<0.001

Data are means (SD) for continuous variables and % for categorical variables

The patients with MS had lower mean serum magnesium (P < 0.001) and a tendency towards higher mean serum PTH (P = 0.067), than those without MS. In contrast, the levels of 25(OH)D did not differ significantly between groups.

### PTH, vitamin D, magnesium, calcium, phosphate and components of MS

PTH was significantly correlated with 25(OH)D (r = -0.29), calcium (r = -0.20), phosphate (r = -0.33) and magnesium (r = 0.17) (all P < 0.001). As shown in table 2, PTH correlated significantly with systolic and diastolic blood pressure (both P < 0.001), and the patients with elevated blood pressure had a significantly higher mean PTH-concentration than those with normal blood pressure; 5.9 (2.4) vs. 5.5 (2.2) pmol/l (P = 0.005). Finally, 25(OH)D correlated significantly with HDL-cholesterol and triglycerides (both P < 0.001), and magnesium correlated significantly with fasting glucose and insulin resistance (both P < 0.001) (table 2).

**Table 2:** Correlations between PTH, 25(OH)D, magnesium and individual components of metabolic syndrome including insulin resistance (HOMA-IR)

	Fasting Glucose (mmol/l)	Systolic Blood Pressure (mm Hg)	Diastolic Blood Pressure (mm Hg)	HDL-Cholesterol (mmol/l)	Triglycerides (mmol/l)	HOMA-Insulin Resistance
	r	r	r	r	r	r
	P	P	P	P	P	P

PTH	0.02	0.15	0.14	0.05	-0.01	-0.06
(n = 1015)	0.477	<0.001	<0.001	0.092	0.752	0.133
						
25(OH)D	-0.04	-0.05	-0.04	0.13	-0.12	-0.09
(n = 973)	0.189	0.139	0.239	<0.001	<0.001	0.009
						
Magnesium	-0.18	-0.05	-0.05	0.04	-0.10	-0.14
(n = 1016)	<0.001	0.135	0.111	0.223	0.001	<0.001

Spearman's rank correlation coefficient (r), P = significance (2-tailed).

### PTH, vitamin D, magnesium and odds for having MS

**Figure 1:**
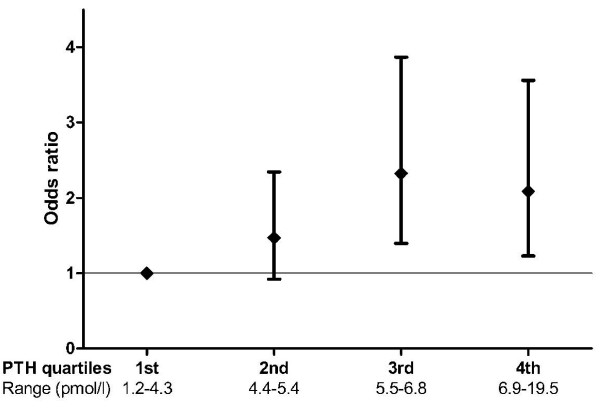
**Odds ratios (95% CI) for metabolic syndrome by quartiles of PTH (reference quartile 1 = 1) after adjustment for 25(OH)D (ln), magnesium, calcium, phosphate, creatinine, age, gender, season, BMI, current smoking, albuminuria, CRP, HOMA-IR and type 2 diabetes (yes/no) (Model 3)**.

Increasing PTH levels were associated with significantly higher odds for having MS in all three multiple logistic regression models (table 3). Higher magnesium levels were significantly associated with lower odds of MS in model 1 and 2, but not after the final adjustment for diabetes (model 3). No statistically significant interaction was observed between gender and PTH (Model 3; P = 0.104). 25(OH)D was not significantly associated with MS in the analyses. To explore a potential non-linear relationship between MS and PTH, quartiles of PTH were implemented in an additional analysis (model 3). This analysis confirmed an independent effect of PTH on the occurrence of MS, showing that patients with PTH levels in the second to fourth quartiles had a 1.5- to 2-fold increased odds of MS (1^st ^quartile reference, P for trend 0.008) (figure 1). The latter findings were consistent after the exclusion of patients using thiazides, loop diuretics, ACE-inhibitors, angiotensin II type 1 receptor blockers, and vitamin D supplements (data not shown).

**Table 3:** Odds for prevalent metabolic syndrome according to levels of PTH, 25(OH)D and magnesium adjusted for confounding factors

	Model 1		Model 2		Model 3	
Explanatory variables	OR (95% CI)	P	OR (95% CI)	P	OR (95% CI)	P
PTH	1.69 (1.12–2.56)	0.013	2.25 (1.34–3.78)	0.002	2.62 (1.52–4.53)	0.001
25(OH)D	0.86 (0.62–1.20)	0.383	0.96 (0.65–1.44)	0.860	1.06 (0.69–1.63)	0.783
Magnesium	0.02 (0.00–0.15)	<0.001	0.04 (0.00–0.47)	0.010	0.22 (0.02–2.54)	0.224

Model 1. Explanatory variables in the multiple logistic regression model were PTH (ln), 25(OH)D (ln), magnesium, calcium, phosphate, and creatinine.

Model 2. Explanatory variables as in model 1 plus age, gender, season, BMI, current smoking, albuminuria, CRP. and HOMA-IR.

Model 3. Explanatory variables as in model 2 plus type 2 diabetes (yes/no).

### Metabolic syndrome-presence or absence of type 2 diabetes?

The above results indicate that PTH and magnesium might associate differently to MS depending on the presence or absence of diabetes. We therefore arranged the patients in three groups: patients without MS, patients with MS but without type 2 diabetes, and patients with both MS and type 2 diabetes (Table 4). Patients with MS but not type 2 diabetes had significantly higher PTH concentrations than patients without MS, whereas patients with both MS and type 2 diabetes had significantly lower serum magnesium concentrations than those without MS.

**Table 4:** Characteristics according to the metabolic syndrome and type 2 diabetes

Variables	Metabolic syndrome absent	Metabolic syndrome present	
		Type 2 diabetes absent	Type 2 diabetes present
Number of patients	323 (32%)	437 (43%)	257 (25%)
PTH (pmol/l)	5.6 (2.3)	6.2 (2.4) *	5.5 (2.2)
25(OH)D (nmol/l)	53 (20)	52 (23)	52 (22)
Magnesium (mmol/l)	0.86 (0.07)	0.85 (0.08)	0.81 (0.08) **

Data are n (%) or means (SD).

Comparison with "metabolic syndrome absent": * P < 0.005 and ** P < 0.001 (ANOVA, Tukeys post hoc)

## Discussion

The major and novel finding of this study is that the serum level of PTH was an independent predictor of MS in a series of 1,017 consecutive morbidly obese women and men. In addition, our data show that morbidly obese patients with both MS and type 2 diabetes were characterized by lower serum magnesium levels as compared to patients without MS.

Although vitamin D status, as assessed by 25(OH)D, was inversely correlated with PTH, we did not find any significant association between serum levels of 25(OH)D and MS.

### PTH, vitamin D and MS

Although several lines of evidence indicate that serum PTH may be associated with metabolic disturbances, to our knowledge, only three recently published studies have addressed the combined effect of serum PTH and vitamin D on MS [8,9,15]. These reports, however, diverge in their conclusions. Our finding of a close relationship between MS and PTH level is in contrast with the negative results of a previous study of severely obese subjects [15], but extend the results from a study of older mainly non-obese men [9] to be valid in adult Caucasian treatment seeking morbidly obese women and men. Further, although we found a significant inverse correlation between 25(OH)D and PTH, we could not confirm any association between 25(OH)D and MS as shown by others [8].

### Magnesium, PTH, MS and diabetes

Low serum magnesium has been shown to predict type 2 diabetes [11] and MS [7]. Our study partly supports these observations, as increasing serum magnesium was associated with lower odds for MS (model 1 and 2). In addition, we document that patients with both type 2 diabetes and MS had lower magnesium levels than patients without MS. However, after the final adjustment for diabetes (model 3), serum magnesium was not significantly associated with MS. The apparent paradoxically low mean serum PTH concentration in patients with both MS and type 2 diabetes could be partly explained by the concomitant low magnesium level, as low serum magnesium is known to inhibit PTH secretion [12].

### What explains the possible link between PTH and MS?

The recently published link between PTH and MS in older men, was explained by insulin resistance, high blood pressure, hyperglycemia and low HDL-cholesterol [8,9]. The hypothesis that PTH may be involved in the pathogenesis of hypertension is also supported by a prospective population based cohort study [22]. We confirm a positive correlation between serum PTH and blood pressure, but we could not verify any correlations between PTH and insulin resistance [23], blood glucose or blood lipids [8,9].

Obesity, older age, and reduced daily intakes of calcium and vitamin D are associated with higher levels of PTH. Accordingly, weight reduction and higher intakes of calcium and vitamin D have been associated with decreases in PTH levels [24,25]. However, whether lowering of serum PTH translates into beneficial effects on MS or its components remains unclear.

### Strengths and limitations

The validity of our findings is strengthened by the prospective collection and registration of data from a homogenous large population of morbidly obese individuals. In addition, the relationship between PTH and MS was robust after adjustment for possible confounders including age, gender, BMI, serum calcium, phosphate, magnesium, vitamin D levels, insulin resistance and type 2 diabetes.

Our study also had limitations. First, the cross-sectional design makes it difficult to establish a cause-effect relationship. Second, our results may not be valid in non-white populations. Third, the internal validity of the study is restricted by the biochemical analyses performed at a routine basis, thus increasing the risk of type 2 errors. Finally, we cannot exclude the possibility that referral of patients to a tertiary care center might have introduced a selection bias.

## Conclusion

We found that PTH was an independent predictor of MS in a large cohort of treatment seeking morbidly obese Caucasian women and men. If our findings are confirmed, randomized intervention trials employing different strategies to lower PTH (e.g. dietary modulation or weight reduction), with improvements in the components of MS as primary endpoints, should be considered.

## Abbreviations

(MS): The metabolic syndrome; (25 [OH]D): 25-hydroxyvitamin D; (PTH): Parathyroid hormone.

## Competing interests

The authors declare that they have no competing interests.

## Authors' contributions

JH contributed to the conception and design, acquisition of data, statistical analysis and interpretation of data, drafted the manuscript and revised it critically for important intellectual content. DH contributed to the conception and design, acquisition of data, interpretation of data, drafted the manuscript and revised it critically for important intellectual content. ETA contributed to interpretation of data, was involved in drafting the manuscript and revised it critically for important intellectual content. TJ contributed to interpretation of data, was involved in drafting the manuscript and revised it critically for important intellectual content. JM contributed to interpretation of data, was involved in drafting the manuscript and revised it critically for important intellectual content. HH contributed to interpretation of data, was involved in drafting the manuscript and revised it critically for important intellectual content. JR contributed to the statistical analyses, interpretation of data, was involved in drafting the manuscript and revised it critically for important intellectual content. JB contributed to interpretation of data, was involved in drafting the manuscript and revised it critically for important intellectual content. All authors read and approved the final manuscript.
